# Mental Health of Children With Special Educational Needs and the Return to In-Person Learning After the COVID-19 Pandemic

**DOI:** 10.1001/jamanetworkopen.2023.46106

**Published:** 2023-12-07

**Authors:** Winnie Wan Yee Tso, Lok Kan Leung, Mathew Siu Chun Chow, Yuliang Wang, Cuixin Li, Ka Yi Hui, Lawrence Ma, Mei Wa Wong, Michael Pak Kiu Lui, Wilfred Hing Sang Wong, So Lun Lee, Tatia Mei Chun Lee, Patrick Ip

**Affiliations:** 1 Department of Paediatrics and Adolescent Medicine, The University of Hong Kong, Pokfulam, Hong Kong; 2 State Key Laboratory of Brain and Cognitive Sciences, The University of Hong Kong, Pokfulam, Hong Kong

## Abstract

**Importance:**

There is limited evidence on whether the quality of life and behavior of children with special educational needs (SEN) have improved or worsened since schools reopened after COVID-19–related school closures.

**Objective:**

To describe the changes in the mental well-being of children and adolescents with SEN during the initial 6 months of resuming in-person learning after COVID-19–related school closures.

**Design, Setting, and Participants:**

This repeated cross-sectional study reported data from surveys completed by parents and caregivers of children and adolescents aged 3 to 18 years with SEN studying at special schools in Hong Kong. The first cohort was obtained during COVID-19–related school closure in April 2020 (wave 1) and the second cohort was obtained 6 months after school resumption with data collection between July and October 2021 (wave 2). Data analysis occurred from January to June 2022.

**Exposure:**

Diagnosis of a disability or disorder that required school-based special educational programming.

**Main Outcomes and Measures:**

Children’s emotional and behavioral difficulties (measured with the Strengths and Difficulties Questionnaire [SDQ]), quality of life (measured with the Pediatric Quality of Life Inventory [PedsQL]), lifestyle habits, parental stress, and parental well-being (measured with the PedsQL Family Impact Module) were assessed. Cross-sectional comparisons of well-being between the 2 waves were conducted using analysis of covariance, and multiple regression analysis was performed to identify factors associated with mental health outcomes in wave 2.

**Results:**

In wave 1, a total of 456 parents and caregivers of children with SEN (mean [SD] age, 7.44 [3.98] years; 315 boys [69.1%]; 141 girls [30.9%]) responded to the surveys. In wave 2, 519 parents and caregivers of children with SEN (mean [SD] age, 8.16 [4.47] years; 365 boys [70.3%]; 154 girls [29.7%]) responded. After school resumption, preschoolers aged 3 to 5 years with SEN had significantly fewer emotional difficulties (mean [SD] SDQ score, 3.26 [2.39] vs 2.68 [2.03]; standardized mean difference [SMD] = 0.26; 95% CI, 0.07-0.46; Bonferroni-corrected *P* = .04) and conduct difficulties (mean [SD] SDQ score, 2.88 [1.89] vs 2.41 [1.91]; SMD = 0.25; 95% CI, 0.05-0.44; Bonferroni-corrected *P* = .01), whereas adolescents had more conduct difficulties (mean [SD] SDQ score, 1.62 [1.50] vs 2.37 [3.02]; SMD = 0.41; 95% CI, 0.13-0.70; Bonferroni-corrected *P* = .049). The overall quality of life of school-aged children with SEN aged 6 to 11 years worsened after school resumption (mean [SD] PedsQL score, 67.52 [17.45] vs 60.57 [16.52]; SMD = 0.41; 95% CI, 0.19-0.62; Bonferroni-corrected *P* = .002).

**Conclusions and Relevance:**

The findings of this repeated cross-sectional study suggest that preschoolers with SEN had improved emotional and behavioral functioning when school resumed after COVID-19–related closures. School-aged children with SEN, adolescents with SEN, and children with intellectual disabilities were at risk of reduced quality of life, indicating that additional support should be offered to vulnerable groups as they return to schools.

## Key Points

**Question:**

How did the mental well-being of children with special educational needs (SEN) change when schools reopened after the COVID-19 pandemic?

**Findings:**

In this repeated cross-sectional study comparing the mental well-being of 456 children with SEN during school closure with 519 children with SEN after school reopening, preschoolers had fewer behavioral and emotional difficulties after school resumption, but school-aged children, adolescents, and children with intellectual disabilities experienced more emotional and behavioral difficulties and deteriorated quality of life.

**Meaning:**

These findings suggest that the mental health outcomes varied according to the age and disabilities of children with SEN after school resumption, indicating that vulnerable groups might require additional support in the school setting.

## Teaser

This cross-sectional study analyzes changes in mental well-being of children with special educational needs (SEN) after returning to in-person learning after COVID-19–related school closures.

## Introduction

Children and adolescents in all countries are continuing to experience the negative consequences of the COVID-19 pandemic. Children with special educational needs (SEN) are one of the most vulnerable groups because they require additional support. Disruptions to daily routines can cause anxiety, frustration, and other negative behaviors^1,2,3^ in children with SEN. During the pandemic, children with SEN were found to have poorer mental health and quality of life and were at higher risk of maltreatment.^4^ School closures and lockdown measures contributed substantially to the negative consequences on children’s mental and physical health and family life. In fact, the COVID-19 pandemic has resulted in the largest disruption of education systems worldwide, affecting nearly 1.6 billion students in more than 190 countries.^5^

In ordinary times, schools provide an education to children in a safe environment, as well as other resources, including free and nutritious school meals for children from low-income families. Schools also allow interpersonal and social interactions, provide occupational opportunities, and offer access to equipment, space, and time for physical activities.^6^ In particular, children with SEN also receive additional support at schools including rehabilitation training and therapy.

During the COVID-19 pandemic, Hong Kong was one of the first cities to mandate school closures to minimize the spread of the virus. Between January 2020 and March 2021, children in Hong Kong did not attend school in person for more than 15 months.^7,8,9,10,11,12,13,14,15,16,17,18^ During school closures, remote learning and online schooling were widely implemented, but it was reported that children with mental health conditions and developmental delays or disorders faced many difficulties.^19,20^ Children with SEN were reported to have poorer mental well-being and their parents had significantly higher parental stress compared with children without SEN.^1^ Some studies^4,21^ highlighted that school closures throughout the course of the pandemic not only affected the health of children with SEN, but also the well-being of all children across all social groups.

In March 2021, schools gradually reopened in Hong Kong, which was a great relief for many parents and children. Although return to school would allow resumption of regular intensive rehabilitation training, it is expected that children with disabilities or SEN would still require more time to adjust and adapt to environmental changes. A survey^22^ conducted after schools reopened showed that children with emotional difficulties had increased self-reported difficulties, whereas parent-rated mental health measures showed no changes, which suggests these children experienced greater stresses in adjusting to school life following recurrent lockdowns and school closures. However, it is still unclear how children with SEN might cope with returning to school and what problems they might encounter when readapting to face-to-face teaching. This repeated cross-sectional study investigated whether postpandemic school resumption was associated with the mental well-being of children with SEN. We aimed to identify potential factors associated with poorer well-being in children with SEN as they return to school in the postpandemic era.

## Methods

### Study Design and Participants

This repeated cross-sectional study was approved by the University of Hong Kong and Hospital Authority Hong Kong West Cluster institutional review board. The study followed the Strengthening the Reporting of Observational Studies in Epidemiology (STROBE) reporting guideline.^23^ The study included parents and their children (aged 3 to 18 years) with SEN who were enrolled at special schools and special childcare centers. In Hong Kong, children with SEN diagnosed by pediatricians and clinical psychologists attend special childcare centers from ages 3 to 6 years and then attend special schools until age 18 years.

The first wave of data collection was conducted in April 2020 during the pandemic when schools were closed by the Education Bureau.^10^ The second wave was conducted 15 months later from July to October 2021 after face-to-face teaching resumed in all schools and childcare centers in early April 2021.^12^ Stratified sampling was used to recruit children aged 6 to 18 years attending special schools across difference districts in Hong Kong. For preschool children aged 3 to less than 6 years, they were recruited from the 2 largest service providers with special childcare centers throughout the territories in Hong Kong. After obtaining written informed consent, parents completed either an online questionnaire or a paper-based questionnaire via invitations from their children’s corresponding institutions. Children who had not started school yet or who were outside the target age range were excluded from the data analyses. Duplicate entries were also removed.

### Measures

Locally validated scales were adopted to examine the physical and mental well-being of children and parents during and after school closures (eAppendix in Supplement 1). Children’s emotional and behavioral difficulties were assessed using the Strengths and Difficulties Questionnaire (SDQ),^24^ and quality of life was measured using the Pediatric Quality of Life Inventory (PedsQL).^25^ Questions on the SDQ are scored from 0 to 2, with a higher total score indicating more difficulty in a given domain. Scores on the PedsQL range from 0 to 100, with a higher score indicating a better quality of life. Parents also responded to survey questions about their child’s lifestyle habits, such as sleep duration, physical activities, and use of electronic devices.

Parents’ well-being was measured using the PedsQL Family Impact Module,^26^ and parental stress was measured by the Chinese Parental Stress Scale.^27^ Family demographics including children’s age, gender, and family socioeconomic status were obtained. A family socioeconomic status index was extracted from principal component analysis,^28^ with factors including household income per capita, parents’ education level, employment, marital status, and whether the family was financially supported by the government under the Comprehensive Social Security Assistance Scheme. The first factor was extracted to estimate the family socioeconomic status index. Parents were also asked to report whether any clinic, rehabilitation and medical appointments, or surgical procedures had been canceled due to the pandemic. All the scales used in the study were parent-proxy questionnaires.

### Statistical Analysis

Descriptive statistics were used to analyze family demographics and the measured variables. Univariate analysis of covariance was conducted to compare SDQ, PedsQL (Generic Core and Family Impact Module), Chinese Parental Stress Scale scores, and children’s lifestyle habits, between the 2 waves, adjusting for children’s age, gender, and socioeconomic status. The χ^2^ goodness of fit test was conducted to examine any changes in the access to health care services (as categorical variables) before and after school closures.

Mediated multiple regression was performed to examine the association of children’s social and behavioral difficulties with quality of life and parental well-being to investigate the factors associated with poorer health outcomes after school resumption in wave 2. In the hierarchical multiple regression, the direct association of emotional and behavioral difficulties (SDQ) with quality of life (PedsQL) was examined, whereas the indirect associations were investigated using parental well-being as the mediator. Children’s age, gender, and family socioeconomic status were also controlled in the regression model. A subcohort of participants who filled in the questionnaires in both waves were extracted to compare the longitudinal changes in children’s and parent’s well-being between the 2 time points by paired sample *t* test. A 2-sided *P* < .05 was considered statistically significant. All statistics were performed using SPSS statistical software version 28 (IBM). The PROCESS macro version 4.2 for SPSS was used for the mediated regression analysis.^29^ Data analysis was conducted from January to June 2022.

## Results

In the first wave in 2020 during school closure, 1088 parents were reached, and 456 parents (41.9%) completed the questionnaires. In wave 2 after school resumption, 1294 parents were reached, and 519 parents (40.1%) responded. This resulted in a sample of 456 children (mean [SD] age, 7.44 [3.98] years; 315 boys [69.1%]; 141 girls [30.9%]) in wave 1 and 519 children (mean [SD] age, 8.16 [4.47] years; 365 boys [70.3%]; 154 girls [29.7%]) in wave 2 (Table). As schools reopened during the second wave, there were fewer disruptions to medical appointments and rehabilitation training among children with SEN compared with the first wave during school closures (Table). After schools reopened, children with SEN did not show significant changes to their emotional and behavioral difficulties, but they had worse prosocial behavior in comparison with the period during school closures (mean [SD] SDQ score, 5.98 [2.23] vs 5.34 [2.55]; standardized mean difference [SMD], 0.26; 95% CI, 0.14-0.40; *P* < .001; Bonferroni-corrected *P* = .001). Children’s quality of life was significantly worse with lower scores for overall quality of life (mean [SD] PedsQL score, 66.32 [18.29] vs 63.78 [17.29]; SMD, 0.14; 95% CI, 0.02-0.27; *P* = .03, Bonferroni-corrected *P* = .14), emotional functioning (mean [SD] PedsQL score, 73.14 [18.31] vs 69.79 [17.26]; SMD, 0.19; 95% CI, 0.06-0.32; *P* = .002; Bonferroni-corrected *P* = .01), and psychosocial functioning (mean [SD] PedsQL score, 64.76 [17.77] vs 61.30 [17.12]; SMD, 0.19; 95% CI, 0.07-0.33, *P* = .006; Bonferroni-corrected *P* = .03). Regarding lifestyle habits, children with SEN generally spent less time on electronic devices, except for internet surfing and social networking. Parents’ well-being and family functioning remained stable between the 2 waves, but parents reported more worries about their children’s disability management as reflected by a lower score in the PedsQL Family Impact Module (mean [SD] score, 58.84 [23.24] vs 54.67 [22.34]; SMD, 0.18; 95% CI, 0.06-0.31; *P* = .008; Bonferroni-corrected *P* = .04).

**Table.** Characteristics and Well-Being Profile of Children With Special Educational Needs Between the 2 Waves

Characteristic	Children, No. (%) (N = 456)		*P* value^a^	Adjusted *P* value^b^	SMD (95% CI)^c^
	Wave 1 (n = 456)	Wave 2 (n = 519)			
Gender					
Male	315 (69.1)	365 (70.3)	.68	NA	0.027 (−0.100 to 0.150)
Female	141 (30.9)	154 (29.7)			
Age, mean (SD), y	7.44 (3.98)	8.16 (4.47)	.008	NA	−0.168 (−0.290 to −.042)
Age group, y					
3-5	198 (43.4)	222 (42.8)	.01	NA	NA
6-11	174 (38.2)	163 (31.4)			
12-18^d^	84 (18.4)	134 (25.8)			
Socioeconomic status index, mean (SD)	0.07 (0.97)	−0.06 (1.02)	.06	NA	0.126 (−0.010 to 0.260)
Type of disability					
Physical	107 (23.5)	105 (20.2)	NA	NA	NA
Intellectual	183 (40.1)	227 (43.7)			
Visual	39 (8.6)	26 (5.0)			
Hearing	26 (5.7)	29 (5.6)			
Psychiatric illness and mental disorders	200 (43.9)	264 (50.9)			
Other	22 (4.8)	22 (4.2)			
Access to health care services					
Disrupted clinic attendance	236 (52.0)	214 (41.7)	.002	NA	NA
Interrupted rehabilitation training	315 (69.7)	184 (36.3)	<.001	NA	NA
Disrupted medical appointments	268 (59.4)	258 (50.5)	.006	NA	NA
Children’s well-being					
Strengths and Difficulties Questionnaire score, mean (SD)					
Total difficulties	13.72 (6.47)	13.88 (5.90)	.84	>.99	−0.027 (−0.150 to 0.100)
Emotional symptoms	2.90 (2.27)	2.85 (2.15)	.61	>.99	0.020 (−0.110 to 0.150)
Conduct problems	2.42 (1.93)	2.33 (1.92)	.41	>.99	0.046 (−0.080 to 0.170)
Hyperactivity or inattention	5.03 (2.51)	5.21 (2.40)	.17	>.99	−0.073 (−0.200 to 0.060)
Social difficulties	3.80 (2.20)	4.05 (2.12)	.30	>.99	−0.114 (−0.240 to 0.010)
Prosocial behavior	5.98 (2.23)	5.34 (2.55)	<.001	.001	0.266 (0.140 to 0.400)
Externalizing score	7.18 (3.73)	7.24 (3.58)	.72	>.99	−0.017 (−0.140 to 0.110)
Internalizing score	6.61 (3.70)	6.73 (3.38)	.93	>.99	−0.033 (−0.160 to 0.090)
Quality of life (PedsQL) score, mean (SD)					
Overall quality of life	66.32 (18.29)	63.78 (17.29)	.03	.14	0.143 (0.020 to 0.270)
Physical functioning	68.36 (26.69)	66.90 (24.39)	.26	>.99	0.057 (−0.070 to 0.180)
Emotional functioning	73.14 (18.31)	69.79 (17.26)	.002	.01	0.188 (0.060 to 0.320)
Social functioning	56.32 (23.64)	52.81 (23.12)	.08	.42	0.150 (0.020 to 0.280)
Psychosocial functioning	64.76 (17.77)	61.30 (17.12)	.006	.03	0.198 (0.070 to 0.330)
Children’s lifestyle habits					
Physical activities, mean (SD), h	1.12 (1.18)	1.23 (1.03)	.18	NA	−0.100 (−0.230 to 0.030)
Sleep, mean (SD), h	10.48 (1.23)	10.26 (1.32)	.04	NA	0.173 (0.050 to 0.300)
Electronic device use, mean (SD), h					
Television	2.19 (1.97)	1.62 (2.16)	<.001	NA	0.269 (0.100 to 0.440)
Homework	1.23 (2.35)	0.57 (0.81)	<.001	NA	0.443 (0.280 to 0.610)
Internet and social networking sites	0.90 (1.68)	1.26 (2.39)	.05		−0.164 (−0.330 to 0.000)
Gaming	2.08 (2.43)	1.80 (3.99)	.20	NA	0.078 (−0.080 to 0.240)
Parental well-being					
Parental stress, mean (SD) score	53.99 (12.01)	55.06 (12.63)	.29	NA	−0.087 (−0.210 to 0.04)
Quality of life (PedsQL, family impact module) score, mean (SD)					
Emotional functioning	65.11 (22.83)	63.38 (22.06)	.42	>.99	0.077 (−0.060 to 0.210)
Worry	58.84 (23.24)	54.67 (22.34)	.008	.040	0.183 (0.060 to 0.310)
Daily activities	48.64 (22.26)	49.74 (23.33)	.51	>.99	−0.048 (−0.170 to 0.080)
Family relationships	62.95 (20.10)	62.55 (21.44)	.84	>.99	0.019 (−0.110 to 0.150)
Family functioning	57.61 (18.51)	57.76 (19.83)	.88	>.99	−0.008 (−0.140 to 0.120)

Abbreviations: NA, not applicable; PedsQL, Pediatric Quality of Life Inventory; SMD, standardized mean difference.

^a^ Well-being comparison was adjusted for age, gender, and socioeconomic status.

^b^ Adjusted *P* value was determined by Bonferroni corrections.

^c^ SMD was calculated on the basis of nonadjusted mean values.

^d^ The χ^2^ test of age groups showed that there were fewer than expected adolescents (12 to 18 years) in wave 1.

Parents of 36 children with SEN (mean [SD] age, 12.14 [3.98] years; 22 boys [61.1%]; 14 girls [38.9%]) participated in both waves. Longitudinal analysis showed that children had significantly more emotional difficulties after school resumption (mean [SD] SDQ score, 3.17 [2.28] vs 3.87 [2.37]; SMD = −0.50; 95% CI, −0.84 to −0.14; *P* = .006), and spent less time sleeping (mean [SD], 10.62 [1.23] hours vs 9.94 hours; SMD = 0.62; 95% CI, 0.26 to 0.97; *P* < .001) and playing video games (mean [SD], 2.44 [2.50] hours vs 1.31 [1.72] hours; SMD = 0.56; 95% CI, 0.07 to 1.04; *P* = .03) (eTable 1 in Supplement 1) in comparison with wave 1.

**Figure 1.**
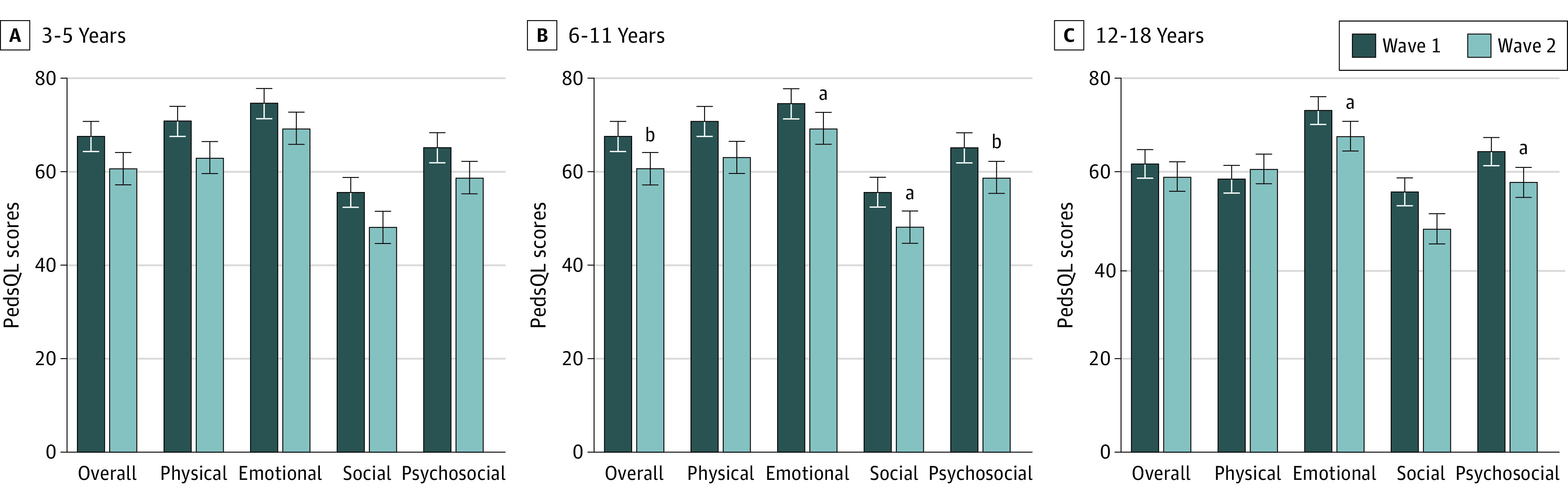
Quality of Life of Children With Special Educational Needs Between 2 Waves by Age Group The figure shows scores from various domains of the Pediatric Quality of Life Inventory (PedsQL) for children ages 3 to 5 years (A), 6 to 11 years (B), and 12 to 18 years (C). Scores on the PedsQL range from 0 to 100, with a higher score indicating a better quality of life. Wave 1 was conducted in April 2020 when schools were closed, and wave 2 was conducted July to October 2021 after in-person learning resumed. Error bars denote SEMs. ^a^Indicates *P* < .01. ^b^Indicates *P* < .001.

Subgroup analysis (Figure 1; eTable 2 and eFigure in Supplement 1) showed that children aged 3 to 5 years had significantly fewer emotional difficulties (mean [SD] SDQ score, 3.26 [2.39] vs 2.68 [2.03]; SMD = 0.26; 95% CI, 0.07-0.46; Bonferroni-corrected *P* = .04) and conduct difficulties (mean [SD] SDQ score, 2.88 [1.89] vs 2.41 [1.91]; SMD = 0.25, 95% CI 0.05-0.44; Bonferroni-corrected *P* = .01) compared with children in wave 1. Only 75 of 222 preschoolers (33.8%) reached the borderline clinical cutoff^30^ for conduct difficulties in wave 2 after school resumption compared with 88 of 198 preschoolers (44.4%) with scores reaching the borderline cutoff in wave 1 during school closure (χ^2^_1420_ = 5.01; *P* = .03). In contrast, children aged 12 to 18 years had significantly increased conduct difficulties (mean [SD] SDQ score, 1.62 [1.50] vs 2.37 [3.02]; SMD = 0.41; 95% CI 0.13-0.70; Bonferroni-corrected *P* = .049) after school resumption compared with children in wave 1. Older children had poorer quality of life after school resumption, with poorer emotional functioning (mean [SD] PedsQL score for children aged 6-11 years, 74.51 [17.53] vs 69.23 [17.07]; SMD = 0.31; 95% CI, 0.09-0.52; Bonferroni-corrected *P* = .02; mean [SD] PedsQL score for children aged 12-18 years, 73.04 [18.82] vs 67.55 [17.98]; SMD = 0.30; 95% CI, 0.02-0.57; Bonferroni-corrected *P* = .03), social functioning (mean [SD] PedsQL score for children aged 6-11 years, 55.54 [22.46] vs 48.08 [21.16]; SMD = 0.34; 95% CI, 0.13-0.56; Bonferroni-corrected *P* = .04), and psychosocial functioning (mean [SD] PedsQL score for children aged 6-11 years, 65.06 [16.41] vs 58.68 [15.78]; SMD = 0.40; 95% CI, 0.18-0.61; Bonferroni-corrected *P* = .004; mean [SD] PedsQL score for children aged 12-18 years, 64.27 [19.33] vs 57.63 [18.44]; SMD = 0.35; 95% CI, 0.08-0.63; Bonferroni-corrected *P* = .045) than children in wave 1. After school resumption, children aged 6 to 11 years were found to have poorer overall quality of life (mean [SD] PedsQL score, 67.52 [17.45] vs 60.57 [16.52]; SMD = 0.41; 95% CI, 0.19-0.62; Bonferroni-corrected *P* = .002) affecting all aspects of their daily functioning compared with children in wave 1. Compared with the quality of life of school-aged children with typical development during the COVID-19–related school closure,^4^ 96 of 153 school-aged children with SEN (58.9%) were found to have poorer quality of life after school resumption (wave 2) with scores that were 1 SD below the scores of typically developing children versus only 70 of 174 children with SEN (40.2%) in wave 1 with quality of life scores 1SD below scores of typically developing children (χ^2^_1337_ = 11.73; *P* < .001).

Regarding lifestyle habits after school resumption, preschool and school-aged children spent significantly less time on electronic devices compared with children in wave 1. Adolescents spent more time surfing the internet, using electronic devices for social networking (mean [SD], 1.27 [2.45] hours vs 2.56 [3.80] hours; SMD =  −0.37; 95% CI, −0.71 to −0.02; *P* = .01) and less time sleeping (mean [SD], 10.16 [1.28] hours vs 9.71 [1.27] hours; SMD = 0.61; 95% CI, 0.33 to 0.90; *P* = .03) in comparison with adolescents in wave 1. In the subgroup analysis comparing parental well-being, parents of school-aged children showed worse emotional functioning (mean [SD] PedsQL family impact score, 67.35 [22.26] vs 61.88 [20.01]; SMD = 0.26; 95% CI, 0.03 to 0.49; Bonferroni-corrected* P* = .04) and more worries (mean [SD] PedsQL family impact score, 61.64 [23.36] vs 50.69 [20.35]; SMD = 0.50; 95% CI, 0.28 to 0.72; Bonferroni-corrected *P* < .001) after school resumption, in comparison with the parents of children in wave 1.

When stratifying by disability type, in comparison with children with intellectual disabilities in wave 1, children in wave 2 had significantly lower scores in their emotional functioning (mean [SD] PedsQL score, 73.93 [18.75] vs 68.12 [18.91]; SMD = 0.31; 95% CI, 0.11-0.51; Bonferroni-corrected *P* = .03) (eTable 3 in Supplement 1). Moreover, parents reported more worries about their children with intellectual disabilities (mean [SD] PedsQL score, 58.52 [23.74] vs 49.98; SMD = 0.37; 95% CI, 0.18-0.57; Bonferroni-corrected *P* = .02) or multiple disabilities (mean [SD] PedsQL score, 57.36 [23.17] vs 48.11 [22.56]; SMD = 0.41; 95% CI, 0.21-0.60; Bonferroni-corrected* P* = .04) after school resumption, and parents of children with psychiatric illness or mental disorders reported higher levels of caregiving stress (mean [SD] PedsQL score, 55.47 [11.94] vs 57.99 [12.14]; SMD = 0.21; 95% CI, 0.02-0.39; *P* = .049) in comparison with parents of children with intellectual disability or multiple disabilities and psychiatric illness or mental disorders in wave 1.

**Figure 2.**
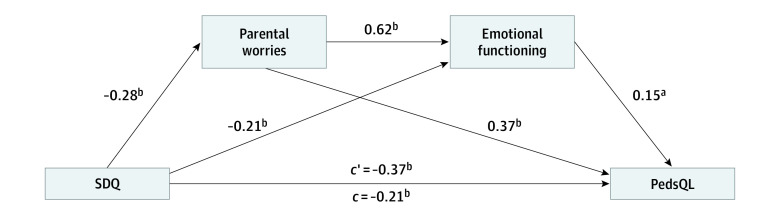
Serial Mediation Analysis Between Children’s Behavioral Outcomes and Quality of Life With Parental Worries and Emotional Functioning as Mediators in Wave 2 The diagram shows the mediation analysis of the association of children’s behavioral outcomes as measured by the Strengths and Difficulties Questionnaire (SDQ) with quality of life as measured by the Pediatric Quality of Life Inventory (PedsQL) in which parental worries and children’s emotional functioning serve as mediators of the association. The variable *c*′ indicates the direct association, and *c* indicates the total association. ^a^Indicates *P* < .01. ^b^Indicates *P* < .001.

The mediated regression analysis was conducted with SDQ as the independent variable; PedsQL as the criterion; parental worries and emotional functioning as the mediators; and age, gender, and socioeconomic status as the covariates (Figure 2). The analysis revealed that children’s emotional and behavioral difficulties had a significant negative association with quality of life, which was partially mediated by parental worries and emotional functioning (total association, β = −0.37; 95% CI, −1.33 to −0.85; *P* < .001; direct association, β = −0.21; 95% CI, −0.85 to −0.40; *P* < .001). Pathways analysis by bootstrapping test using 10 000 samples and 95% CIs revealed 3 significant pathways: (1) children’s behavioral difficulties were associated with parental worries and parental worries were associated with children’s quality of life (indirect association, β = −0.10; 95% CI, −0.15 to −0.06), (2) children’s behavioral difficulties were associated with parental emotions and parental emotions were associated with children’s quality of life (indirect association, β = −0.03; 95% CI, −0.06 to −0.01, and (3) children’s behavioral difficulties were associated with parental worries and parental emotions which were associated with children’s quality of life (indirect association, β = −0.03; 95% CI, −0.05 to −0.01). The direct association model explained 44.7% of the variance of the outcome (*R*^2^ = 0.248; *F*_4,441_ = 36.29; *P* < .001), whereas adding the 2 mediators into the model resulted in an *R*^2^ change of 19.9% (total association model, *R*^2^ = 0.447, *F*_6,439_ = 59.07; *P* < .001).

## Discussion

To our knowledge, this cross-sectional study is among the first to describe the changes in the mental well-being of children with SEN after school resumption in the COVID-19 era. Our findings showed the associations of school resumption with the well-being and behavior of children with SEN varied according to their age group. A previous study^31^ on the impact of the pandemic lockdown also found variations between different subgroups. In terms of support for children with SEN, Ghandour et al^32^ found that children with special needs had distinct challenges in accessing appropriate care, especially older children and children with high support needs, which might be associated with potential underlying inequalities.

Our study showed that preschoolers with SEN showed significant improvements in emotional difficulties and conduct difficulties. Preschoolers did not have deterioration in their quality of life after school resumption. The improvements in preschoolers’ emotional and behavioral difficulties soon after school resumption might be related to the higher neuroplasticity of younger brains allowing them to respond better and faster to the face-to-face lessons and resumed rehabilitation training. However, it is worth noting that preschoolers had deteriorated prosocial behavior, likely due to the lack of social interactions with peers during school closures and lockdowns. Parents and early childhood educators should encourage group play and activities that promote social interactions in preschoolers with SEN.

Unlike the younger age group, school-aged children (6-11 years) with SEN had poorer quality of life in all domains after school resumption, and adolescents’ quality of life also deteriorated but to a lesser extent. The association of school resumption with quality of life in older children is likely multifactorial. With prolonged school closures and lack of rehabilitation training, children with SEN lost access to resources that help maintain their overall physical functioning and mental health. Moreover, children with attention-deficits, intellectual disability, and visual or physical impairments might have difficulty in accessing online learning during school closures. With schools reopening, these children may struggle to meet the academic demands after prolonged periods away from school. In particular, children with poorer social and communication skills such as those with autism would have missed out on socializing and behavioral training during school closures, leading to difficulties in readapting to the school environment and interacting with their peers.

After school resumption, school-aged children with SEN had poorer quality of life in comparison with the children with SEN during school closure. Our results showed that this deterioration might not be directly associated with worsened emotional or behavioral functioning in school-aged children; rather, we found that worsened emotional and behavioral functioning in children were associated with increased parental worry and poorer emotional functioning. Parental worry and poorer emotional functioning were associated with poorer quality of life in children. Of the 3 age groups, only parents of school-aged children with SEN had significantly reduced emotional functioning and increased worry. Moreover, some children with SEN were in the middle of transitioning from the preschool service or kindergarten to the special schools during the school closures. Parents expressed substantial worries that their children might not receive sufficient rehabilitation training due to the pandemic prior to entering the special schools. Although remote schooling was provided during school closures, school-aged children with SEN had substantial difficulties and barriers to accessing online learning.^33^ Changes to the provision of schooling and educational settings may cause substantial parental worries; hence, parental support during this vulnerable period is crucial.

Adolescents with SEN showed more emotional difficulties and conduct problems after school resumption. Adolescents might have more challenging behaviors that could lead to poorer family relationships. Because adolescents with SEN might be struggling at school and at home, their quality of life would deteriorate after school resumption. For the adolescent age group, support should focus on interventions that can improve adolescents’ emotional symptoms and externalizing behaviors.

We found that children with intellectual disabilities were the most vulnerable group with deteriorated quality of life that affected their daily functioning (eg, hyperactivity, externalizing behaviors, and reduced prosocial behaviors). Parents and caregivers of children with mental disabilities such as autism and attention-deficit/hyperactivity disorders also showed significantly increased parental stress and worries after school resumption. These findings were consistent with our previous study^1^ that showed children with intellectual disabilities or mental disorders had significantly more emotional and behavioral difficulties and poorer quality of life compared with children with other disabilities.

### Strengths and Limitations

This study had a number of important strengths. This is one of very few studies that specifically investigated a cohort of children with SEN. We used a repeated cross-sectional design rather than a retrospective cohort to infer changes. Because parental factors are associated with the well-being of children with SEN, we used a survey approach rather than analyzing population-based data of children with SEN, which allows us to understand parental stress, emotions, and worries.

This study also had some limitations. First, this was a survey-based study, which might be prone to recall and reporting bias. Nevertheless, because the first data collection phase (wave 1) was done during territory-wide school closures with a stringent social distancing policy due to the COVID-19 pandemic, an electronic survey method was considered to be the safest and most effective way to collect data from a large number of subjects. The study might be prone to proxy-report bias because we invited parents and caregivers to report their child’s or adolescent’s well-being only. Nevertheless, because many participants were either young children, school-aged children, or adolescents with intellectual disabilities, it would be difficult to obtain reliable self-reported measures. Second, many parents of children with SEN were reluctant to disclose the identity of their children and preferred to give anonymous data due to possible stigmatization, which meant we could only collect longitudinal data from 36 children with SEN over the 2 waves. Nevertheless, we managed to recruit additional participants from the same network of special schools as in the first wave. In addition, the subgroup analysis of the longitudinal data from the first wave showed similar findings to that of the repeated cross-sectional data. Third, although the overall response rates were 41.9% in wave 1 and 40.1% in wave 2, the types and proportions of disabilities among the children recruited in both wave 1 and wave 2 were similar to the types and proportions of disabilities of all children attending special schools in Hong Kong according to statistics from the Government of the Hong Kong Special Administrative Region.^34^ Hence, we believed we had a representative sample and our findings could be generalized to the overall population with SEN. Third, as this is a repeated cross-sectional survey, we cannot draw any conclusions on the associations. Furthermore, other unaccounted factors could be responsible for the observed results, such as the association of post–COVID-19 condition with the mental well-being of children or the effects of the global recession.

## Conclusions

During the first 6 months of schools reopening after the COVID-19 pandemic, school-aged children and adolescents with SEN, including children with intellectual disabilities and psychiatric illness, had a higher risk of deteriorated quality of life. Although preschool children did not show significantly reduced well-being, they were found to have worse prosocial behaviors. Parents or caregivers of children with psychiatric illness were also at risk of increased parental stress and worries. In the postpandemic era, there is no doubt that early school resumption was beneficial for all children; however, our study highlighted that specific groups of children and adolescents with SEN were vulnerable to poorer mental well-being during the early phase of school resumption. Educators, health care workers, and policymakers should provide additional support to vulnerable children and their parents. As the impacts varied according to age group and type of disability, personalized support with a focus on rehabilitation is needed for children with SEN.

## Notes

Supplement 1. **eAppendix.** Description of Well-Being Outcome Measures **eTable 1.** Longitudinal Comparison of 36 Subjects Across the Two Waves **eTable 2.** Well-Being Profile of Children With Special Educational Needs by Age Group Between Two Waves **eFigure.** Well-Being Profile of Children With Special Educational Needs and Their Parents Between Two Waves by Age Groups **eTable 3.** Well-Being Profile of Children With Special Educational Needs by Different Disabilities

Supplement 2.
Data Sharing Statement
