# Supplementary material for: Mental Health of Children With Special Educational Needs and the Return to In-Person Learning After the COVID-19 Pandemic
Source: JAMA Netw Open. 2023 Dec 7;6(12):e2346106. doi: 10.1001/jamanetworkopen.2023.46106 (PMC10704277; doi:10.1001/jamanetworkopen.2023.46106)
Supplement: Supplement 1. — eAppendix. Description of Well-Being Outcome Measures eTable 1. Longitudinal Comparison of 36 Subjects Across the Two Waves eTable 2. Well-Being Profile of Children With Special Educational Needs by Age Group Between Two Waves eFigure. Well-Being Profile of Children With Special Educational Needs and Their Parents Between Two Waves by Age Groups eTable 3. Well-Being Profile of Children With Special Educational Needs by Different Disabilities [file jamanetwopen-e2346106-s001.pdf]

# Supplemental Online Content

Tso WWY, Leung LK, Chow MSC, et al. Mental health of children with special educational needs and the return to in-person learning after the COVID-19 pandemic. J*AMA Netw Open*. 2023;6(12):e2346106. doi:10.1001/jamanetworkopen.2023.46106

**eAppendix.** Description of Well-Being Outcome Measures

**eTable 1.** Longitudinal Comparison of 36 Subjects Across the Two Waves

**eTable 2.** Well-Being Profile of Children With Special Educational Needs by Age Group Between Two Waves

**eFigure.** Well-Being Profile of Children With Special Educational Needs and Their Parents Between Two Waves by Age Groups

**eTable 3.** Well-Being Profile of Children With Special Educational Needs by Different Disabilities

This supplemental material has been provided by the authors to give readers additional information about their work.

© 2023 Tso et al. *JAMA Network Open.*

eAppendix. Descriptions of well-being outcome measures

The Strengths and Difficulties Questionnaire (SDQ) is a 25-item 3-point Likert scale questionnaire measuring children's emotional and behavioural difficulties[1]. There are 5 subscales in SDQ, including emotional symptoms, conduct problems, attention/ hyperactivity difficulties, peer problems and prosocial behavior respectively. A higher score in SDQ total difficulties score, as well as the subscale scores in emotional symptoms, conduct problems, attention/ hyperactivity difficulties and peer problems indicate more difficulties of children, while a lower score in prosocial behavior subscale indicates worse performance.

The Pediatric Quality of Life Inventory (PedsQL) was adopted to measure children's quality of life[2]. The PedsQL Generic Core Scale consists of 23 items assessing their physical (8 items), emotional (5 items), social (5 items) and school functioning (5 items), with scores ranging from 0-100 and a higher score indicates better quality of life and functioning. Children's psychosocial functioning is calculated by the means of emotional and social functioning. In this study, 18 items were adopted as school functioning subscale was not assessed due to school closures in wave 1 April 2020 without any face-to-face lessons.

The PedsQL Family Impact Module consists of 36 items measuring parental well-being and family functioning[3]. Parental well-being includes physical (6 items), emotional (5 items), social (4 items), and cognitive functioning (5 items), communication (3 items), and worry (5 items), while family functioning includes family daily activities (3 items) and family relationships (5 items). In this study 18 items were adopted to investigate parents' emotional functioning, worry and family functioning including daily activities and family relationships. The scores range from 0-100, with a higher score indicate better functioning.

The Chinese Parental Stress Scale (PSS) is a 17-item 6-point Likert scale measuring parental stress[4]. A higher total score indicates a higher level of parental stress.

References


1. Lai KY, Luk ES, Leung PW, Wong AS, Law L, Ho K. Validation of the Chinese version of the strengths and difficulties questionnaire in Hong Kong. *Soc Psychiatry Psychiatr Epidemiol*. 2010;45(12):1179-1186.
2. Chan LFP, Chow SMK, Lo SK. Preliminary validation of the Chinese version of the Pediatric Quality of Life Inventory. *Int J Rehabil Res*. 2005;28(3):219-227.
3. Varni JW, Sherman SA, Burwinkle TM, Dickinson PE, Dixon P. The PedsQL Family Impact Module: preliminary reliability and validity. *Health Qual Life Outcomes*. 2004;2:55. doi:10.1186/1477-7525-2-55
4. Leung C, Tsang SK. The Chinese parental stress scale: psychometric evidence using Rasch modeling on clinical and nonclinical samples. *J Pers Assess*. 2010;92(1):26-34.


© 2023 Tso et al. *JAMA Network Open.*

eTable 1. Longitudinal Comparison of 36 Subjects Across the Two Waves.

| | Wave 1 Mean (SD) | Wave 2 Mean (SD) | *P* value |
|---|---|---|---|
| Children's well-being | | | |
| Behavioural outcomes (SDQ) | | | |
| Total difficulties | 14.10 (6.02) | 14.74 (5.68) | .34 |
| Emotional symptoms | 3.17 (2.28) | 3.87 (2.37) | .006** |
| Conduct problems | 1.97 (1.51) | 1.88 (1.49) | .86 |
| Hyperactivity/ inattention | 4.57 (2.27) | 5.11 (2.38) | .14 |
| Peer problems | 4.46 (2.57) | 4.14 (2.38) | .45 |
| Prosocial behaviour | 5.94 (2.20) | 5.64 (2.05) | .47 |
| Externalising score | 6.39 (2.70) | 6.66 (3.16) | .46 |
| Internalising score | 7.56 (4.12) | 7.87 (3.69) | .50 |
| Quality of life (PedsQL) | | | |
| Overall quality of life | 60.38 (22.12) | 55.91 (19.55) | .09 |
| Physical functioning | 63.37 (30.89) | 57.20 (29.69) | .09 |
| Emotional functioning | 69.72 (20.84) | 66.08 (14.93) | .20 |
| Social functioning | 46.25 (25.05) | 43.75 (21.63) | .46 |
| Psychosocial functioning | 57.99 (20.81) | 54.85 (16.21) | .22 |
| | | | |
| Children's lifestyle habits | | | |
| Physical activities, hours | 0.98 (0.82) | 0.96 (0.87) | .84 |
| Sleep, hours | 10.62 (1.23) | 9.94 (1.14) | .001** |
| Electronic devices, hours | | | |
| TV | 1.29 (2.4) | 2.4 (5.15) | .35 |
| Homework | 1.19 (1.41) | 0.58 (0.55) | .33 |
| Internet & SNS | 0.86 (2.0) | 1.23 (1.52) | .29 |
| Gaming | 2.44 (2.5) | 1.31 (1.72) | .026* |
| | | | |
| Parental well-being | | | |
| Parental stress | 52.79 (12.83) | 52.79 (12.83) | .09 |
| | | | |
| PedsQL family impact | | | |
| Parents' emotion | 63.04 (22.50) | 64.31 (21.15) | .78 |
| Worry | 56.11 (24.93) | 54.31 (21.59) | .58 |
| Daily activities | 50.23 (21.50) | 51.85 (21.09) | .58 |
| Family relationships | 64.03 (20.10) | 64.17 (20.2) | .96 |
| Family functioning | 58.84 (18.96) | 59.55 (18.7) | .79 |

* $P < .05$, ** $P < .01$

© 2023 Tso et al. *JAMA Network Open.*

eTable 2. Well-Being Profile of Children With Special Educational Needs by Age Group Between the Two Waves.

A. Pre-school (3-5 years)

| | Wave 1 (N = 198) | Wave 2 (N = 222) | | | |
|---|---|---|---|---|---|
| | Mean/ N (SD/ %) | Mean/ N (SD/ %) | *P* value [a] | Adjusted *P* value | SMD (95% CI) [b] |
| Access to health care services | | | | | |
| Disrupted clinic attendance | 101 (51.3) | 83 (37.9) | .008** | | |
| Interrupted rehabilitation training | 155 (79.1) | 75 (34.7) | <.001*** | | |
| Disrupted medical appointment | 111 (56.9) | 97 (44.5) | .014* | | |
| Children's well-being | | | | | |
| Behavioural outcomes (SDQ) | | | | | |
| Total difficulties | 14.70 (6.40) | 13.79 (5.65) | .036* | .25 | 0.15 (-0.04 to 0.35) |
| Emotional symptoms | 3.26 (2.39) | 2.68 (2.03) | .006** | .042* | 0.26 (0.07 to 0.46) |
| Conduct problems | 2.88 (1.89) | 2.41 (1.91) | .002** | .014* | 0.25 (0.05 to 0.44) |
| Hyperactivity/ inattention | 5.35 (2.52) | 5.43 (2.30) | .75 | 1.00 | -0.03 (-0.23 to 0.16) |
| Peers problems | 3.72 (2.18) | 3.85 (2.03) | .81 | 1.00 | -0.06 (-0.26 to 0.13) |
| Prosocial behaviour | 5.78 (2.08) | 5.08 (2.57) | .02* | .14 | 0.3 (0.1 to 0.49) |
| Externalising score | 7.91 (3.67) | 7.47 (3.48) | .053 | .37 | 0.12 (-0.07 to 0.32) |
| Internalising score | 6.95 (3.74) | 6.42 (3.23) | .07 | .50 | 0.15 (-0.04 to 0.34) |
| Quality of life (PedsQL) | | | | | |
| Overall quality of life | 67.27 (17.52) | 69.06 (15.85) | .23 | 1.00 | -0.11 (-0.3 to 0.09) |
| Physical functioning | 70.56 (23.24) | 73.65 (20.72) | .22 | 1.00 | -0.14 (-0.33 to 0.05) |
| Emotional functioning | 71.96 (18.76) | 71.54 (16.86) | .95 | 1.00 | 0.02 (-0.17 to 0.22) |
| Social functioning | 57.30 (23.81) | 59.35 (21.95) | .17 | .86 | -0.09 (-0.28 to 0.1) |
| Psychosocial functioning | 64.70 (18.30) | 65.44 (16.43) | .43 | 1.00 | -0.04 (-0.24 to 0.15) |
| Children's lifestyle habits | | | | | |
| Physical activities, hours | 1.18 (1.34) | 1.48 (1.07) | .048* | | -0.25 (-0.44 to -0.06) |
| Sleep, hours | 10.76 (1.20) | 10.75 (1.19) | .94 | | 0.31 (0.12 to 0.51) |
| Electronic devices, hours | | | | | |
| TV | 2.35 (1.83) | 1.34 (1.30) | <.001*** | | 0.68 (0.42 to 0.94) |

© 2023 Tso et al. *JAMA Network Open.*

| | | | | | |
|---|---|---|---|---|---|
| Homework | 1.29 (3.25) | 0.40 (0.59) | <.001*** | | 0.46 (0.21 to 0.71) |
| Internet & SNS | 0.66 (1.10) | 0.58 (1.08) | .91 | | 0.07 (-0.19 to 0.32) |
| Gaming | 1.37 (1.93) | 1.35 (3.54) | .49 | | 0.01 (-0.24 to 0.26) |
| Parental well-being | | | | | |
| Parental stress | 54.79 (12.68) | 54.99 (12.59) | .62 | | -0.02 (-0.21 to 0.18) |
| PedsQL family impact | | | | | |
| Parents' emotion | 63.42 (23.44) | 63.97 (22.64) | .31 | 1.00 | -0.02 (-0.22 to 0.17) |
| Worry | 57.28 (23.61) | 58.89 (22.37) | .34 | 1.00 | -0.07 (-0.26 to 0.12) |
| Daily activities | 46.82 (22.46) | 48.50 (23.16) | .23 | 1.00 | -0.07 (-0.27 to 0.12) |
| Family relationships | 60.10 (20.67) | 62.23 (21.85) | .12 | .59 | -0.1 (-0.29 to 0.09) |
| Family functioning | 55.12 (19.41) | 57.08 (20.23) | .12 | .58 | -0.1 (-0.29 to 0.09) |

B. School-aged (6-11 years)

| | Wave 1 (N = 174) | Wave 2 (N = 163) | | | |
|---|---|---|---|---|---|
| | Mean/ N (SD/ %) | Mean/ N (SD/ %) | *P* value[a] | Adjusted *P* value | SMD (95% CI)[b] |
| Access to health care services | | | | | |
| Disrupted clinic attendance | 88 (50.9) | 74 (45.7) | .38 | | |
| Interrupted rehabilitation training | 117 (67.2) | 53 (32.9) | <.001*** | | |
| Disrupted medical appointment | 103 (59.9) | 95 (58.6) | .83 | | |
| Children's well-being | | | | | |
| Behavioural outcomes (SDQ) | | | | | |
| Total difficulties | 13.59 (6.66) | 13.87 (6.03) | .38 | 1.00 | -0.04 (-0.26 to 0.17) |
| Emotional symptoms | 2.67 (2.23) | 2.95 (2.12) | .26 | 1.00 | -0.13 (-0.35 to 0.09) |
| Conduct problems | 2.28 (2.00) | 2.20 (1.90) | .96 | 1.00 | 0.04 (-0.18 to 0.25) |
| Hyperactivity/ inattention | 5.21 (2.40) | 5.20 (2.42) | .34 | 1.00 | 0 (-0.21 to 0.22) |
| Peer problems | 3.87 (2.19) | 4.09 (2.13) | .33 | 1.00 | -0.1 (-0.32 to 0.11) |
| Prosocial behaviour | 6.03 (2.29) | 5.56 (2.44) | .06 | .41 | 0.2 (-0.02 to 0.42) |
| Externalising score | 7.20 (3.79) | 7.09 (3.74) | .63 | 1.00 | 0.03 (-0.19 to 0.24) |
| Internalising score | 6.44 (3.77) | 6.86 (3.40) | .25 | 1.00 | -0.12 (-0.33 to 0.1) |

© 2023 Tso et al. *JAMA Network Open.*

| | | | | | |
|---|---|---|---|---|---|
| Quality of life (PedsQL) | | | | | |
| Overall quality of life | 67.52 (17.45) | 60.57 (16.52) | <.001*** | .002** | 0.41 (0.19 to 0.62) |
| Physical functioning | 70.69 (26.93) | 62.96 (25.13) | .012* | .06 | 0.3 (0.08 to 0.51) |
| Emotional functioning | 74.51 (17.53) | 69.23 (17.07) | .003** | .015* | 0.31 (0.09 to 0.52) |
| Social functioning | 55.54 (22.46) | 48.08 (21.16) | .008** | .04* | 0.34 (0.13 to 0.56) |
| Psychosocial functioning | 65.06 (16.41) | 58.68 (15.78) | <.001*** | .004** | 0.4 (0.18 to 0.61) |
| Children's lifestyle habits | | | | | |
| Physical activities, hours | 1.22 (1.14) | 1.12 (0.91) | .45 | | 0.09 (-0.12 to 0.31) |
| Sleep, hours | 10.31 (1.18) | 10.02 (1.28) | .08 | | 0.53 (0.31 to 0.75) |
| Electronic devices, hours | | | | | |
| TV | 2.13 (1.88) | 1.48 (1.62) | .025* | | 0.38 (0.09 to 0.67) |
| Homework | 1.17 (1.48) | 0.52 (0.63) | <.001*** | | 0.63 (0.35 to 0.9) |
| Internet & SNS | 0.96 (1.66) | 1.03 (1.42) | .95 | | -0.05 (-0.32 to 0.23) |
| Gaming | 2.37 (2.71) | 1.63 (2.81) | .05 | | 0.27 (0.01 to 0.52) |
| Parental well-being | | | | | |
| Parental stress | 53.58 (11.72) | 54.61 (12.03) | .31 | | -0.09 (-0.3 to 0.13) |
| PedsQL family impact | | | | | |
| Parents' emotion | 67.35 (22.26) | 61.88 (20.01) | .007** | .035* | 0.26 (0.03 to 0.49) |
| Worry | 61.64 (23.36) | 50.69 (20.35) | <.001*** | <.001*** | 0.5 (0.28 to 0.72) |
| Daily activities | 49.66 (22.19) | 50.15 (23.34) | .85 | 1.00 | -0.02 (-0.24 to 0.19) |
| Family relationships | 64.45 (20.28) | 63.60 (19.10) | .51 | 1.00 | 0.04 (-0.17 to 0.26) |
| Family functioning | 58.94 (18.01) | 58.58 (17.93) | .58 | 1.00 | 0.02 (-0.19 to 0.23) |

C. Adolescents (12-18 years)

| | Wave 1 (N = 84) | Wave 2 (N = 134) | | | |
|---|---|---|---|---|---|
| | Mean/ N (SD/ %) | Mean/ N (SD/ %) | *P* value[a] | Adjusted *P* value | SMD (95% CI)[b] |
| Access to health care services | | | | | |
| Disrupted clinic attendance | 47 (56.0) | 57 (43.2) | .07 | | |

© 2023 Tso et al. *JAMA Network Open.*

| | | | | | |
|---|---|---|---|---|---|
| Interrupted rehabilitation training | 43 (52.4) | 56 (43.1) | .21 | | |
| Disrupted medical appointment | 54 (64.3) | 66 (50.4) | .05 | | |
| Children’s well-being | | | | | |
| Behavioural outcomes (SDQ) | | | | | |
| Total difficulties | 11.61 (5.74) | 14.06 (6.18) | .033* | .23 | -0.41 (-0.69 to -0.13) |
| Emotional symptoms | 2.50 (1.92) | 3.02 (2.37) | .14 | 1.00 | -0.24 (-0.52 to 0.04) |
| Conduct problems | 1.62 (1.50) | 2.37 (1.99) | .007** | .049* | -0.41 (-0.7 to -0.13) |
| Hyperactivity/ inattention | 3.90 (2.39) | 4.84 (2.52) | .051 | .36 | -0.38 (-0.66 to -0.1) |
| Peer problems | 3.88 (2.27) | 4.35 (2.24) | .54 | 1.00 | -0.21 (-0.49 to 0.07) |
| Prosocial behaviour | 6.39 (2.43) | 5.51 (2.62) | .08 | .56 | 0.34 (0.06 to 0.63) |
| Externalising score | 5.41 (3.12) | 7.04 (3.55) | .008** | .06 | -0.48 (-0.76 to -0.2) |
| Internalising score | 6.15 (3.38) | 7.07 (3.56) | .21 | 1.00 | -0.27 (-0.54 to 0.01) |
| Quality of life (PedsQL) | | | | | |
| Overall quality of life | 61.62 (21.05) | 58.92 (18.22) | .13 | .64 | 0.14 (-0.13 to 0.41) |
| Physical functioning | 58.37 (31.43) | 60.53 (26.43) | .80 | 1.00 | -0.08 (-0.35 to 0.2) |
| Emotional functioning | 73.04 (18.82) | 67.55 (17.98) | .006** | .030* | 0.3 (0.02 to 0.57) |
| Social functioning | 55.63 (25.72) | 47.72 (24.72) | .046* | .23 | 0.32 (0.04 to 0.59) |
| Psychosocial functioning | 64.27 (19.33) | 57.63 (18.44) | .009** | .045* | 0.35 (0.08 to 0.63) |
| Children’s lifestyle habits | | | | | |
| Physical activities, hours | 0.80 (0.71) | 0.96 (1.02) | .31 | | -0.18 (-0.45 to 0.1) |
| Sleep, hours | 10.16 (1.28) | 9.71 (1.27) | .034* | | 0.61 (0.33 to 0.9) |
| Electronic devices, hours | | | | | |
| TV | 1.77 (2.72) | 2.27 (3.40) | .59 | | -0.15 (-0.61 to 0.31) |
| Homework | 1.23 (1.37) | 0.86 (1.15) | .40 | | 0.31 (-0.03 to 0.65) |
| Internet & SNS | 1.27 (2.45) | 2.56 (3.80) | .014* | | -0.37 (-0.71 to -0.02) |
| Gaming | 2.81 (2.40) | 2.73 (5.50) | .83 | | 0.02 (-0.32 to 0.35) |
| Parental well-being | | | | | |
| Parental stress | 52.93 (10.98) | 55.73 (13.45) | .19 | | -0.22 (-0.5 to 0.05) |
| PedsQL family impact | | | | | |

© 2023 Tso et al. *JAMA Network Open.*

| | | | | | |
|---|---|---|---|---|---|
| Parents’ emotion | 65.79 (21.51) | 64.24 (23.53) | .84 | 1.00 | 0.07 (-0.3 to 0.43) |
| Worry | 56.63 (21.74) | 52.50 (23.59) | .22 | 1.00 | 0.18 (-0.1 to 0.46) |
| Daily activities | 50.80 (21.84) | 51.31 (23.65) | .89 | 1.00 | -0.02 (-0.3 to 0.25) |
| Family relationships | 66.48 (17.53) | 61.80 (23.49) | .07 | .34 | 0.22 (-0.06 to 0.5) |
| Family functioning | 60.66 (16.77) | 57.89 (21.45) | .23 | 1.00 | 0.14 (-0.14 to 0.42) |

* *P* < .05, ** *P* < .01, *** *P* < .001

[a] Well-being comparison adjusted for age, gender, and socioeconomic status

[b] SMD is calculated based on non-adjusted mean values.

SMD: Standardized mean difference, CI: confidence interval; SDQ: Strengths and Difficulties Questionnaire; PedsQL: Paediatric Quality of Life; SNS: Social networking sites

Adjusted *P* value is determined by Bonferroni corrections

© 2023 Tso et al. *JAMA Network Open.*

eFigure. Well-Being Profile of Children With Special Educational Needs and Their Parents Between Two Waves by Age Groups *P < .05, **P < .01, *** P < .001, adjusted by Bonferroni corrections

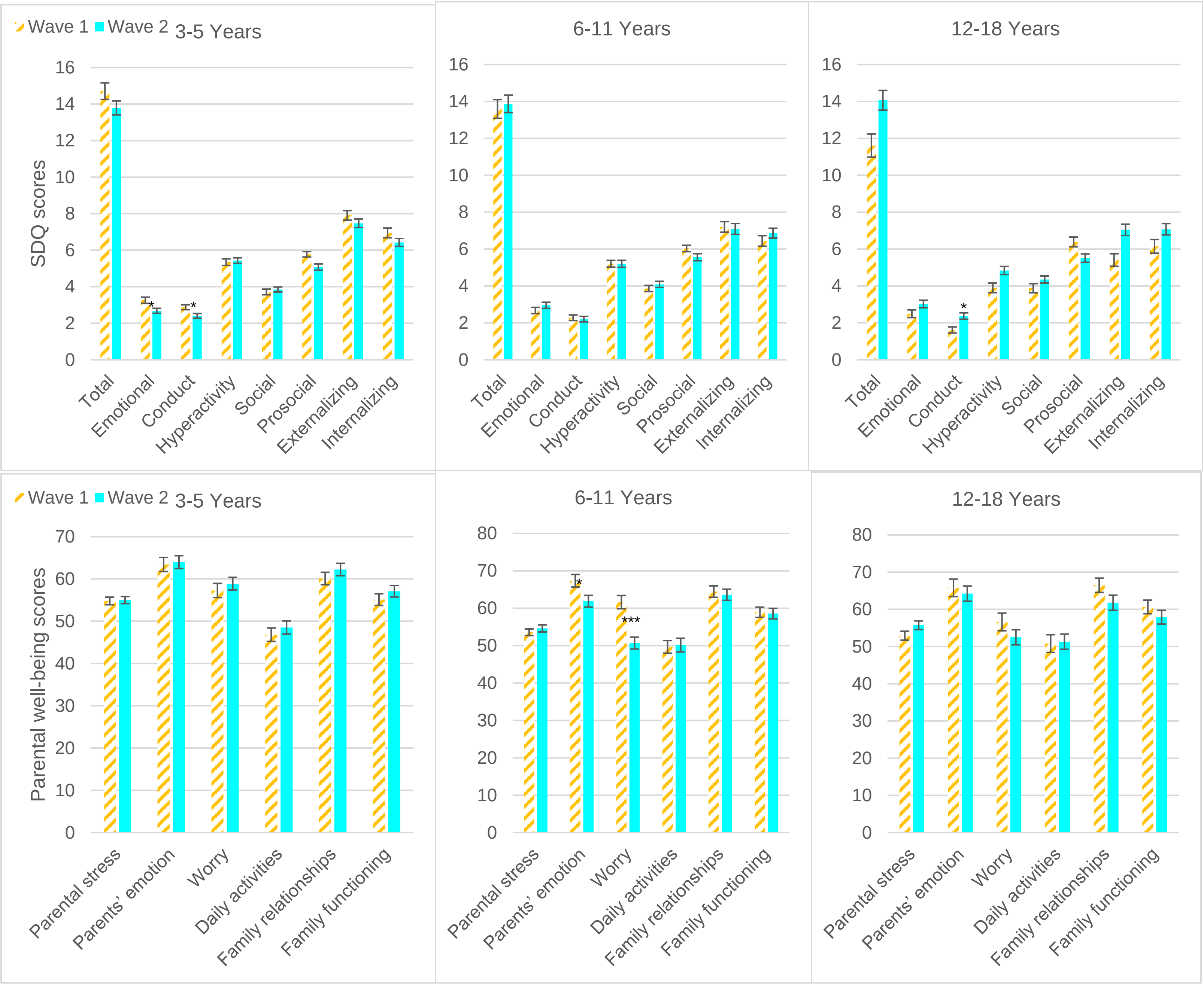


© 2023 Tso et al. *JAMA Network Open.*

eTable 3. Well-Being Profile of Children With Special Educational Needs By Different Disabilities

A. Physical and Visual impairment

| | Physical impairment (N = 212) | | | | Visual impairment (N = 65) | | | |
|---|---|---|---|---|---|---|---|---|
| | Wave 1 (N = 107) Mean/ N (SD/ %) | Wave 2 (N = 105) Mean/ N (SD/ %) | *P* value [a] | Adjusted *P* value | Wave 1 (N = 39) Mean/ N (SD/ %) | Wave 2 (N = 26) Mean/ N (SD/ %) | *P* value [a] | Adjusted *P* value |
| Demographics | | | | | | | | |
| Age | 9.38 (4.56) | 10.5 (4.27) | .07 | | 9.41 (4.2) | 9.58 (4.12) | .88 | |
| Gender | | | .34 | | | | .30 | |
| Male | 57 (53.3) | 63 (60) | | | 21 (53.8) | 18 (69.2) | | |
| Female | 50 (46.7) | 42 (40) | | | 18 (46.2) | 8 (30.8) | | |
| Age group, years | | | .08 | | | | .66 | |
| 3 to 5 | 28 (26.2) | 16 (15.2) | | | 9 (23.1) | 4 (15.4) | | |
| 6 to 11 | 43 (40.2) | 41 (39) | | | 17 (43.6) | 14 (53.8) | | |
| 12 to 18 | 36 (33.6) | 48 (45.7) | | | 13 (33.3) | 8 (30.8) | | |
| Access to healthcare services | | | | | | | | |
| Disrupted clinic attendance | 75 (70.1) | 33 (31.4) | <.001*** | | 29 (74.4) | 11 (42.3) | .018*** | |
| Interrupted rehabilitation training | 74 (69.8) | 39 (37.1) | <.001*** | | 24 (61.5) | 13 (50) | .45 | |
| Disrupted medical appointments | 86 (81.1) | 60 (57.7) | <.001*** | | 31 (81.6) | 14 (53.8) | .026* | |
| Children's well-being | | | | | | | | |
| Behavioural outcomes (SDQ) | | | | | | | | |
| Total difficulties | 11.53 (6.53) | 12.87 (6.04) | .41 | 1.00 | 13.38 (8.22) | 13.45 (7.66) | .85 | 1.00 |
| Emotional symptoms | 2.6 (2.25) | 3.18 (2.03) | .10 | .69 | 3.25 (2.65) | 2.94 (2.7) | .95 | 1.00 |
| Conduct problems | 2.16 (1.88) | 2.17 (2.18) | .81 | 1.00 | 2.77 (2.22) | 1.84 (2.34) | .11 | .76 |
| Hyperactivity/ inattention | 3.84 (2.46) | 4.42 (2.53) | .16 | 1.00 | 4.15 (2.74) | 4.77 (3.05) | .27 | 1.00 |
| Peer problems | 3.1 (1.99) | 3.28 (2.06) | .68 | 1.00 | 3.8 (2.62) | 4.24 (2.32) | .48 | 1.00 |
| Prosocial behaviour | 6.58 (2.4) | 6.31 (2.5) | .51 | 1.00 | 7.01 (2.17) | 4.84 (2.98) | .018* | .13 |
| Externalising score | 5.92 (3.87) | 6.39 (3.99) | .61 | 1.00 | 6.61 (4.16) | 6.4 (5.17) | .95 | 1.00 |
| Internalising score | 5.69 (3.6) | 6.49 (3.38) | .43 | 1.00 | 6.73 (4.56) | 7.01 (4.44) | .68 | 1.00 |

© 2023 Tso et al. *JAMA Network Open.*

| | Physical impairment (N = 212) | | | | Visual impairment (N = 65) | | | |
|---|---|---|---|---|---|---|---|---|
| | Wave 1 (N = 107) Mean/ N (SD/ %) | Wave 2 (N = 105) Mean/ N (SD/ %) | *P* value [a] | Adjusted *P* value | Wave 1 (N = 39) Mean/ N (SD/ %) | Wave 2 (N = 26) Mean/ N (SD/ %) | *P* value [a] | Adjusted *P* value |
| Quality of life (PedsQL) | | | | | | | | |
| Overall quality of life | 51 (17.64) | 49.43 (18.81) | .20 | 1.00 | 54.52 (21.59) | 47.74 (25.87) | .15 | .77 |
| Physical functioning | 37.04 (25.28) | 38.44 (23.83) | .78 | 1.00 | 48.75 (29.08) | 35.25 (31.01) | .023* | .12 |
| Emotional functioning | 72.01 (19.92) | 65.75 (20.67) | .003** | .015* | 68.97 (21.59) | 65.1 (29.39) | .56 | 1.00 |
| Social functioning | 52.33 (26.11) | 50.28 (23.44) | .40 | 1.00 | 49.9 (28.73) | 50.2 (33.15) | .94 | 1.00 |
| Psychosocial functioning | 62.4 (19.41) | 58.08 (19.2) | .033* | .17 | 59.19 (22.07) | 57.84 (26.95) | .78 | 1.00 |
| Children's lifestyle habits | | | | | | | | |
| Physical activities, hours | 1.16 (1.11) | 1.03 (0.88) | .54 | | 1.03 (1.23) | 1.07 (0.98) | .72 | |
| Sleep, hours | 10.67 (1.29) | 10.06 (1.2) | .003** | | 10.57 (1.59) | 10.29 (1.43) | .89 | |
| Electronic devices, hours | | | | | | | | |
| TV | 2.22 (2.23) | 1.95 (1.85) | .66 | | 2.5 (2.4) | 4.23 (7.03) | .86 | |
| Homework | 1.57 (1.49) | 1.04 (1.22) | .015* | | 2.25 (2.22) | 0.62 (0.9) | .015* | |
| Internet & SNS | 1.37 (2.24) | 2.11 (3.35) | .34 | | 1.28 (2.59) | 1.21 (1.93) | .89 | |
| Gaming | 2.16 (2.14) | 2.33 (4.5) | .73 | | 3.05 (4.28) | 0.66 (0.94) | .25 | |
| Parental well-being | | | | | | | | |
| Parental stress | 54.38 (11.83) | 54.12 (13.32) | .56 | | 57.22 (14.7) | 54.99 (16.01) | .41 | |
| PedsQL family impact | | | | | | | | |
| Parents' emotion | 63.86 (21.43) | 60.67 (24.95) | .42 | 1.00 | 55.83 (23.31) | 57.4 (27.81) | .51 | 1.00 |
| Worry | 51.98 (22.18) | 47.12 (23.74) | .05 | .27 | 50.38 (20.79) | 43.8 (24.29) | .45 | 1.00 |
| Daily activities | 44.14 (24.99) | 45.55 (24.06) | .83 | 1.00 | 43.59 (22.17) | 40.33 (25.42) | .60 | 1.00 |
| Family relationships | 60.07 (21.38) | 61.62 (21.09) | .66 | 1.00 | 57.95 (22.53) | 60.2 (19.92) | .50 | 1.00 |
| Family functioning | 54.19 (20.33) | 55.61 (19.08) | .71 | 1.00 | 52.68 (20.07) | 52.75 (19.83) | .85 | 1.00 |

© 2023 Tso et al. *JAMA Network Open.*

B. Hearing impairment and Intellectual disability

| | Hearing impairment (N = 55) | | | | Intellectual disability (N = 410) | | | |
|---|---|---|---|---|---|---|---|---|
| | Wave 1 (N = 26) Mean/ N (SD/ %) | Wave 2 (N =29) Mean/ N (SD/ %) | *P* value [a] | Adjusted *P* value | Wave 1 (N = 183) Mean/ N (SD/ %) | Wave 2 (N = 227) Mean/ N (SD/ %) | *P* value [a] | Adjusted *P* value |
| Demographics | | | | | | | | |
| Age | 7.5 (3.92) | 10.14 (3.98) | .02* | | 8.05 (4.42) | 9.41 (4.47) | .002** | |
| Gender | | | .59 | | | | 1.00 | |
| Male | 15 (57.7) | 19 (65.5) | | | 118 (64.5) | 146 (64.3) | | |
| Female | 11 (42.3) | 10 (34.5) | | | 65 (35.5) | 81 (35.7) | | |
| Age group, years | | | .07 | | | | .012* | |
| 3 to 5 | 10 (38.5) | 4 (13.8) | | | 70 (38.3) | 58 (25.6) | | |
| 6 to 11 | 11 (42.3) | 13 (44.8) | | | 70 (38.3) | 93 (39.8) | | |
| 12 to 18 | 5 (19.2) | 12 (41.4) | | | 43 (23.5) | 76 (33.5) | | |
| Access to healthcare services | | | | | | | | |
| Disrupted clinic attendance | 18 (69.2) | 18 (62.1) | .78 | | 105 (57.7) | 103 (45.6) | .017* | |
| Interrupted rehabilitation training | 18 (72) | 14 (48.3) | 1.00 | | 129 (70.9) | 77 (34.5) | <.001*** | |
| Disrupted medical appointments | 22 (84.6) | 20 (69) | .22 | | 119 (65.7) | 117 (52) | .006** | |
| Children's wellbeing | | | | | | | | |
| Behavioural outcomes (SDQ) | | | | | | | | |
| Total difficulties | 16.43 (7.42) | 12.76 (6.84) | .17 | 1.00 | 13.97 (6.68) | 14.51 (6.09) | .52 | 1.00 |
| Emotional symptoms | 3.64 (2.42) | 2.04 (1.91) | .024* | .17 | 3.02 (2.37) | 2.99 (2.27) | .77 | 1.00 |
| Conduct problems | 3.83 (2.08) | 1.99 (2.34) | .037* | .26 | 2.43 (1.93) | 2.57 (2.14) | .55 | 1.00 |
| Hyperactivity/ inattention | 5.39 (2.47) | 5.09 (2.7) | .91 | 1.00 | 4.91 (2.66) | 5.3 (2.42) | .041* | .29 |
| Peer problems | 3.82 (2.22) | 4.28 (2.41) | .77 | 1.00 | 4 (2.29) | 4.27 (2.14) | .83 | 1.00 |
| Prosocial behaviour | 6.39 (2.2) | 5.73 (2.92) | .32 | 1.00 | 5.94 (2.28) | 5.2 (2.54) | .016* | .11 |
| Externalising score | 8.92 (4.06) | 6.88 (4.61) | .28 | 1.00 | 7.14 (3.86) | 7.6 (3.84) | .17 | 1.00 |
| Internalising score | 7.63 (4.22) | 6.01 (3.44) | .19 | 1.00 | 6.87 (3.82) | 7.02 (3.39) | .92 | 1.00 |
| Quality of life (PedsQL) | | | | | | | | |

© 2023 Tso et al. *JAMA Network Open.*

| | Hearing impairment (N = 55) | | | | Intellectual disability (N = 410) | | | |
|---|---|---|---|---|---|---|---|---|
| | Wave 1 (N = 26) Mean/ N (SD/ %) | Wave 2 (N =29) Mean/ N (SD/ %) | *P* value [a] | Adjusted *P* value | Wave 1 (N = 183) Mean/ N (SD/ %) | Wave 2 (N = 227) Mean/ N (SD/ %) | *P* value [a] | Adjusted *P* value |
| Overall quality of life | 62.53 (24.99) | 55.36 (22.95) | .16 | .78 | 65.19 (19.33) | 59.3 (18.58) | .022* | .11 |
| Physical functioning | 61.66 (30.33) | 51.51 (28.96) | .09 | .46 | 67.65 (25.71) | 61.33 (26.1) | .047* | .24 |
| Emotional functioning | 68.85 (21.74) | 68.97 (23.58) | .89 | 1.00 | 73.93 (18.75) | 68.12 (18.91) | .006** | .03* |
| Social functioning | 57.88 (29.54) | 47.72 (25.32) | .13 | .67 | 52.63 (25.83) | 47.27 (24.7) | .41 | 1.00 |
| Psychosocial functioning | 63.29 (24.08) | 58.43 (21.39) | .34 | 1.00 | 63.23 (19.06) | 57.7 (18.45) | .06 | .29 |
| Children's lifestyle habits | | | | | | | | |
| Physical activities, hours | 0.73 (0.81) | 1.23 (1.1) | .034* | | 1.07 (1) | 1.14 (1.03) | .33 | |
| Sleep, hours | 10.21 (1.43) | 10.27 (1.15) | .12 | | 10.45 (1.34) | 10.22 (1.38) | .48 | |
| Electronic devices, hours | | | | | | | | |
| TV | 2.5 (2.99) | 1.95 (2.3) | .16 | | 2.05 (1.91) | 1.81 (2.8) | .14 | |
| Homework | 0.74 (0.65) | 0.84 (0.86) | .80 | | 1.16 (3.38) | 0.53 (0.74) | .019* | |
| Internet & SNS | 0.66 (1.09) | 1.5 (1.92) | .50 | | 0.87 (1.87) | 1.5 (2.7) | .21 | |
| Gaming | 1.58 (1.35) | 1.49 (1.84) | .48 | | 1.77 (2.18) | 1.67 (4.41) | .68 | |
| Parental well-being | | | | | | | | |
| Parental stress | 54.31 (11.39) | 54.61 (14.81) | .64 | | 55.63 (12.34) | 56.28 (12.43) | .79 | |
| PedsQL family impact | | | | | | | | |
| Parents' emotion | 56.76 (27.33) | 61.03 (24.91) | .97 | 1.00 | 62.79 (22.99) | 61.44 (21.76) | .85 | 1.00 |
| Worry | 50.38 (24.16) | 48.28 (21.1) | .63 | 1.00 | 58.52 (23.74) | 49.98 (22.28) | .004** | .02* |
| Daily activities | 47.76 (21.29) | 46.7 (24.18) | .52 | 1.00 | 48.26 (21.76) | 49.39 (23.2) | .54 | 1.00 |
| Family relationships | 59.62 (19.13) | 65.13 (23.69) | .80 | 1.00 | 62.22 (20.35) | 62.37 (21.69) | .64 | 1.00 |
| Family functioning | 55.17 (17.79) | 58.26 (21.39) | .91 | 1.00 | 57.03 (18.36) | 57.53 (19.58) | .56 | 1.00 |

© 2023 Tso et al. *JAMA Network Open.*

C. Psychiatric illness and Multiple disabilities

| | Psychiatric illness (N = 464) | | | | Multiple disabilities (N = 237) | | | |
|---|---|---|---|---|---|---|---|---|
| | Wave 1 (N = 200) Mean/ N (SD/ %) | Wave 2 (N = 264) Mean/ N (SD/ %) | *P* value [a] | Adjusted *P* value | Wave 1 (N = 160) Mean/ N (SD/ %) | Wave 2 (N = 177) Mean/ N (SD/ %) | *P* value [a] | Adjusted *P* value |
| Demographics | | | | | | | | |
| Age | 6.89 (3.36) | 7.81 (4.42) | .01* | | 7.68 (4.22) | 9.2 (4.57) | .002** | |
| Gender | | | .81 | | | | .91 | |
| Male | 164 (82) | 214 (81.1) | | | 108 (67.5) | 121 (68.4) | | |
| Female | 36 (18) | 50 (18.9) | | | 52 (32.5) | 56 (31.6) | | |
| Age group, years | | | .006** | | | | .015* | |
| 3 to 5 | 95 (47.5) | 127 (48.1) | | | 64 (40) | 51 (28.8) | | |
| 6 to 11 | 81 (40.5) | 79 (29.9) | | | 65 (40.6) | 69 (39) | | |
| 12 to 18 | 24 (12) | 58 (22) | | | 31 (19.4) | 57 (32.2) | | |
| Access to healthcare services | | | | | | | | |
| Disrupted clinic attendance | 94 (47) | 130 (49.6) | .64 | | 95 (59.7) | 83 (47.2) | .022* | |
| Interrupted rehabilitation training | 151 (75.9) | 104 (40.2) | <.001*** | | 119 (75.3) | 64 (36.8) | <.001*** | |
| Disrupted medical appointments | 102 (51.5) | 145 (55.1) | .45 | | 108 (68.4) | 98 (56) | .024* | |
| Children's wellbeing | | | | | | | | |
| Behavioural outcomes (SDQ) | | | | | | | | |
| Total difficulties | 15.51 (6.01) | 15.42 (5.87) | .78 | 1.00 | 14.2 (6.69) | 14.86 (6.25) | .66 | 1.00 |
| Emotional symptoms | 2.97 (2.24) | 3 (2.19) | .80 | 1.00 | 2.98 (2.34) | 3.08 (2.31) | .95 | 1.00 |
| Conduct problems | 2.67 (2.04) | 2.61 (1.96) | .92 | 1.00 | 2.58 (1.98) | 2.61 (2.27) | .98 | 1.00 |
| Hyperactivity/ inattention | 6.05 (2.26) | 5.92 (2.45) | .70 | 1.00 | 5.15 (2.68) | 5.42 (2.52) | .25 | 1.00 |
| Peer problems | 4.52 (2.1) | 4.64 (2.1) | .94 | 1.00 | 4 (2.29) | 4.33 (2.19) | .84 | 1.00 |
| Prosocial behaviour | 5.25 (1.98) | 4.56 (2.52) | .002** | .014* | 5.79 (2.23) | 5.11 (2.7) | .17 | 1.00 |
| Externalising score | 8.27 (3.53) | 8.15 (3.56) | .98 | 1.00 | 7.5 (3.95) | 7.74 (3.96) | .59 | 1.00 |
| Internalising score | 7.33 (3.53) | 7.39 (3.38) | .78 | 1.00 | 6.78 (3.76) | 7.21 (3.51) | .80 | 1.00 |
| Quality of life (PedsQL) | | | | | | | | |

© 2023 Tso et al. *JAMA Network Open.*

| | Psychiatric illness (N = 464) | | | | Multiple disabilities (N = 237) | | | |
|---|---|---|---|---|---|---|---|---|
| | Wave 1 (N = 200) Mean/ N (SD/ %) | Wave 2 (N = 264) Mean/ N (SD/ %) | *P* value [a] | Adjusted *P* value | Wave 1 (N = 160) Mean/ N (SD/ %) | Wave 2 (N = 177) Mean/ N (SD/ %) | *P* value [a] | Adjusted *P* value |
| Overall quality of life | 68.7 (14.8) | 65.38 (15.47) | .08* | .40 | 64.18 (19.32) | 58.24 (19.23) | .04 | .22 |
| Physical functioning | 77.88 (17.81) | 74.99 (19.15) | .21 | 1.00 | 65.86 (25.64) | 60.47 (27.13) | .13 | .64 |
| Emotional functioning | 70.33 (17.3) | 67.59 (17.35) | .21 | 1.00 | 73.4 (18.95) | 66.34 (19.82) | .013* | .065 |
| Social functioning | 52.39 (21.66) | 47.8 (21.69) | .10 | .49 | 52.46 (25.9) | 46.58 (24.83) | .29 | 1.00 |
| Psychosocial functioning | 61.36 (16.06) | 57.69 (16.37) | .07 | .37 | 62.84 (19.17) | 56.47 (19.02) | .05 | .26 |
| | | | | | | | | |
| Children's lifestyle habits | | | | | | | | |
| Physical activities, hours | 1.15 (1.36) | 1.31 (1.05) | .13 | | 1.06 (1.01) | 1.15 (1) | .25 | |
| Sleep, hours | 10.31 (1.2) | 10 (1.28) | .03* | | 10.52 (1.32) | 10.07 (1.35) | .044* | |
| Electronic devices, hours | | | | | | | | |
| TV | 1.91 (1.79) | 1.55 (2.47) | .12 | | 2.12 (1.91) | 1.78 (3.03) | .12 | |
| Homework | 0.96 (1.37) | 0.46 (0.61) | <.001*** | | 1.35 (3.65) | 0.55 (0.79) | .013* | |
| Internet & SNS | 0.69 (1.41) | 1.16 (2.25) | .10 | | 0.66 (1.48) | 1.58 (2.82) | .11 | |
| Gaming | 2.15 (2.59) | 2.05 (4.71) | .67 | | 1.46 (1.85) | 1.86 (5) | .80 | |
| | | | | | | | | |
| Parental well-being | | | | | | | | |
| Parental stress | 55.47 (11.94) | 57.99 (12.14) | .049* | | 56.55 (12.24) | 56.87 (12.95) | .68 | |
| | | | | | | | | |
| PedsQL family impact | | | | | | | | |
| Parents' emotion | 62.81 (23.07) | 60.56 (21.63) | .22 | 1.00 | 62.71 (23.28) | 60 (22.05) | .69 | 1.00 |
| Worry | 56.75 (22.49) | 53.11 (21.17) | .07 | .33 | 57.36 (23.17) | 48.11 (22.56) | .007** | .035 |
| Daily activities | 48.28 (20.81) | 47.2 (22.81) | .73 | 1.00 | 47.43 (21.54) | 47.02 (23.6) | .60 | 1.00 |
| Family relationships | 62.19 (18.48) | 59.32 (22.39) | .17 | .85 | 60.84 (19.81) | 61.76 (22.73) | .21 | 1.00 |
| Family functioning | 56.97 (16.96) | 54.8 (20.32) | .28 | 1.00 | 55.86 (17.76) | 56.24 (20.26) | .28 | 1.00 |

* $P < .05$, ** $P < .01$, *** $P < .001$

[a] Well-being comparison adjusted for age, gender, and socioeconomic status

SDQ: Strengths and Difficulties Questionnaire; PedsQL: Paediatric Quality of Life; SNS: Social networking site

Adjusted *P* value is determined by Bonferroni corrections.

© 2023 Tso et al. *JAMA Network Open.*
