# Supplementary material for: Mental Health of Children With Special Educational Needs and the Return to In-Person Learning After the COVID-19 Pandemic
Source: JAMA Netw Open. 2023 Dec 7;6(12):e2346106. doi: 10.1001/jamanetworkopen.2023.46106 (PMC10704277; doi:10.1001/jamanetworkopen.2023.46106)
Supplement: Supplement 2. — Data Sharing Statement [file jamanetwopen-e2346106-s002.pdf]

Data Sharing Statement

Tso. Mental Health of Children With Special Educational Needs and the Return to In-Person Learning After the COVID-19 Pandemic. *JAMA Netw Open*. Published December 07, 2023. doi:10.1001/jamanetworkopen.2023.46106

Data
Data available: No
